# Editorial: Words and emotions

**DOI:** 10.3389/fpsyg.2024.1486294

**Published:** 2024-10-15

**Authors:** Elham Abdullah Ghobain, Hassan Saleh Mahdi, Haifa Al-Nofaie

**Affiliations:** ^1^Foreign Languages Department, Jazan University, Jizan, Saudi Arabia; ^2^Faculty of Language Studies, Arab Open University, Riyadh, Saudi Arabia; ^3^Foreign Languages Department, Taif University, Taif, Saudi Arabia

**Keywords:** socio-cognitive frameworks, therapeutic communication, emoji and personality traits, sport fanaticism, early language acquisition, sensitive language, visual metaphors in sadness

This Research Topic emerged from a growing recognition of the intricate relationship between language and emotion—a relationship that spans various disciplines and offers profound insights into human behavior and cognition. As researchers in psychology, linguistics, and related fields increasingly focus on the intersection of these domains, it became evident that there is a need for a comprehensive exploration of how language both reflects and shapes emotional experiences. The motivation behind this Research Topic is to bring together cutting-edge research that addresses these dynamics through diverse methodologies, providing a holistic view of how language and emotion influence one another.

The dynamic interplay between language and emotion has been a focal point of research across multiple disciplines, revealing that these two domains are deeply interconnected. Emotional states impact language processing—affecting comprehension, word retrieval, and sentence construction (Barrett, 2017; Pavlenko, 2014). Conversely, language shapes and constructs emotional experiences, providing a conceptual framework for individuals to categorize and articulate their feelings (Lindquist et al., 2012). This intricate relationship has captivated scholars across psychology, linguistics, neuroscience, and media studies, leading to the development of theoretical frameworks that explore the mutual influence between language and emotion. For example, the James-Lange Theory of Emotion examines how bodily responses relate to emotional experiences, while Cognitive Appraisal Theory explores how cognitive evaluations shape emotions. These theories have significantly advanced our understanding of the bidirectional influence between emotions and language, especially in terms of production and comprehension (Ellsworth and Scherer, 2003).

Recent research continues to open new pathways for exploration, leading to the evolution of models in this area. Early work, such as Gross's process model of emotion regulation (Gross, 1998), illustrates how emotional regulation strategies can shape linguistic choices, particularly in social and educational contexts. Technological advancements have further enhanced our ability to analyze language through emotional and psychological lenses. Innovations in psycholinguistics and computational linguistics have introduced sophisticated methods for classifying and assessing language within emotional frameworks, providing invaluable insights into how emotions influence communication across various contexts (Pennebaker, 2011). These evolving theoretical frameworks have uncovered subtle variations in emotional expression related to attention, emotional states, social dynamics, cognitive processes, and individual differences, reshaping linguistic and emotional research.

As the study of language and emotion continues to reveal new layers of complexity, this Research Topic offers a timely exploration of these intersections. The contributions featured in this Research Topic examine themes such as emotional semantics, cross-linguistic emotional experiences, and the neural representation of language. Drawing from diverse disciplinary perspectives, these articles highlight the nuanced and multifaceted relationship between words and emotions, providing readers with fresh insights into this rapidly evolving field. Wen et al. examine the socio-cognitive aspects of visual metaphors in Chinese poetry comics, shedding light on how these metaphors reflect cultural understandings of sadness. Meanwhile, Healey et al. investigate power dynamics in organizational settings, revealing that those in positions of authority tend to avoid sensitive language, complicating assumptions about power and communication. Mobarki and Alzahrani turn their focus to the metaphorical framing of sports fanaticism as a disease in Saudi newspapers, emphasizing the role of language in shaping societal perceptions. Kennison et al. explore emoji usage on social media, finding that personality traits and language patterns can be inferred from how emojis are used, offering new insights into digital communication. Bailey et al. contribute an acoustic analysis of therapy sessions, identifying vocal patterns tied to self-compassion, self-criticism, and self-protection, thereby advancing the understanding of emotional expression in therapeutic contexts. Finally, Zhang et al. examine the use of intonation words in Mandarin-speaking children, demonstrating how these serve as precursors to syntactic development in early language acquisition.

The methodological contributions of these articles further illuminate the issue by employing diverse and innovative approaches to their respective research questions. Wen et al. utilize a socio-cognitive framework and qualitative content analysis to explore visual metaphors in poetry comics, revealing culturally nuanced understandings of sadness. Healey et al. leverage a large corpus of organizational emails and standardized linguistic measures to analyze sensitive language use, offering a new perspective on the interplay between power and communication. Mobarki and Alzahrani's use of a corpus-based approach and metaphor-led discourse analysis provides valuable insights into societal constructions of sports fanaticism through disease metaphors. Kennison et al.'s integration of surveys and computational text analysis to study emoji use and personality traits adds depth to our understanding of digital communication patterns. Bailey et al.'s acoustic analysis of therapy session recordings advances the exploration of vocal expressions of emotion in therapeutic contexts. Lastly, Zhang et al.'s examination of intonation words in early Mandarin-speaking children contributes to the study of language acquisition by linking prosodic features to syntactic development. Each study's methodological approach enriches our understanding of the complex relationship between language and emotion, highlighting the innovative ways in which these phenomena can be studied and interpreted.

Collectively, these articles enhance our understanding of the intricate intersections between language and emotion, offering valuable insights from diverse cultural, organizational, and developmental contexts. Each study contributes uniquely to our comprehension of these complex interactions, advancing knowledge in fields such as language teaching, emotional regulation, and beyond. It is anticipated that the findings presented in this Research Topic will inspire future research and potentially influence practices across linguistics and psychology, fostering a deeper exploration of how language shapes and reflects emotional experiences.
